# The Impact of Arthroscopy on Surgical Decision-Making and Outcomes in Osteoarthritis Patients Undergoing Unicompartmental Knee Arthroplasty

**DOI:** 10.7759/cureus.46684

**Published:** 2023-10-08

**Authors:** Shaohua Liang, Haiquan Zeng, Ming YU, Yang Liu, Wen Wang

**Affiliations:** 1 Orthopedics and Sports Medicine, Guangzhou Red Cross Hospital of Jinan University, Guangzhou, CHN; 2 Orthopedics, Guizhou Medical University, Guiyang, CHN; 3 Orthopedics, Suining Central Hospital, Suining, CHN

**Keywords:** quality of life, knee joint function, unicompartmental knee arthroplasty, arthroscopy, oseoarthritis

## Abstract

Background and objective

Although unicompartmental knee arthroplasty (UKA) is a minimally invasive procedure, its application is limited due to strict criteria related to indications. In clinical practice, the aid of procedures such as arthroscopy is occasionally required to determine the surgical indication and thereby improve prognosis. In light of this, this study aimed to evaluate the impact of intraoperative arthroscopy on surgical decision-making in osteoarthritis (OA) patients and the prognosis of patients undergoing UKA.

Methodology

The clinical records of patients diagnosed with knee OA who underwent knee arthroplasty between January 2017 and January 2020 were retrospectively analyzed. The inclusion criteria were as follows: patients with radiographic evidence of single-compartmental Kellgren-Lawrence (KL) grade 3 or 4 knee OA but presenting symptoms of persistent multicompartmental knee pain or locking for at least six months, with a history of anterior cruciate ligament (ACL) injury or meniscus tear. They had undergone either UKA or total knee arthroplasty (TKA). Data on clinical characteristics and outcomes at baseline and during follow-up were collected.

Results

A total of 429 patients were included in the study. Patients who underwent arthroscopy were more likely to undergo UKA surgery than those who did not (p<0.05). Among patients who underwent UKA, no instances of blood transfusion during hospitalization or postoperative complications were reported, regardless of whether arthroscopy was performed or not. Although the overall Western Ontario and McMaster Universities Osteoarthritis Index (WOMAC) scores and Knee Society Functional Score (KSFS) did not differ between the two groups, the Knee Society Score (KSS) was significantly higher in patients who underwent arthroscopy (88.77 ±5.09) compared to those who did not (85.53 ±5.11). Similarly, the arthroscopy group had a higher overall Forgotten Joint Score (FJS) (44.6 ±4.20) than the UKA-only group (42.05 ±3.58).

Conclusion

Arthroscopy findings can assist in surgical decision-making for OA patients. Performing arthroscopy and UKA simultaneously is relatively safe and may be associated with favorable outcomes.

## Introduction

Osteoarthritis (OA) is a degenerative disease that predominantly affects the knee, resulting in persistent pain, disability, and reduced quality of life [1]. While early-stage knee OA can be managed with conservative measures, surgical intervention is required for patients in the advanced stages. Total knee arthroplasty (TKA) and unicompartmental knee arthroplasty (UKA) are the commonly utilized surgical approaches for OA [2]. Compared to TKA, UKA is a less invasive and more affordable procedure, with comparable long-term prognoses and outcomes [3,4]. However, the choice of surgery might vary among surgeons when patients present with symptoms and signs suggesting multicompartment degeneration.

The selection of the surgical approach for knee OA depends on the patient’s symptoms as well as physical examination and radiographic findings. Isolated anteromedial OA or spontaneous osteonecrosis of the knee are considered the classic indications for UKA [5]. However, the indications for UKA have expanded with advancements in technologies such as prosthesis designs and specialized procedures [6]. Arthroscopy plays a crucial role in the comprehensive management of OA patients, from diagnosis to therapy selection and surgery [7]. It provides valuable insights into the severity and complexity of intra-articular lesions and assists in determining the appropriateness of UKA. Arthroscopy also aids in performing UKA in patients with anterior cruciate ligament (ACL) deficiency and OA [8,9], potentially increasing the likelihood of such patients undergoing UKA, along with influencing long-term outcomes. In this study, we aimed to evaluate the impact of intraoperative arthroscopy on surgical decision-making regarding UKA and assess the difference in therapeutic effects in patients undergoing UKA.

## Materials and methods

Inclusion and exclusion criteria

The clinical records of patients who were diagnosed with knee OA and underwent knee arthroplasty between January 2017 and January 2020 were retrospectively analyzed. The inclusion criteria included persistent multicompartmental knee pain or locking for at least six months, with a history of ACL injury or meniscus tear, and radiographic evidence of single compartmental Kellgren-Lawrence (KL) grade 3 or 4 knee OA. Patients with post-traumatic arthritis, rheumatoid arthritis, and osteonecrosis, those who underwent revision arthroplasty, and patients who dropped out during the two-year follow-up were excluded from the study.

Surgical technique

**Figure 1:**
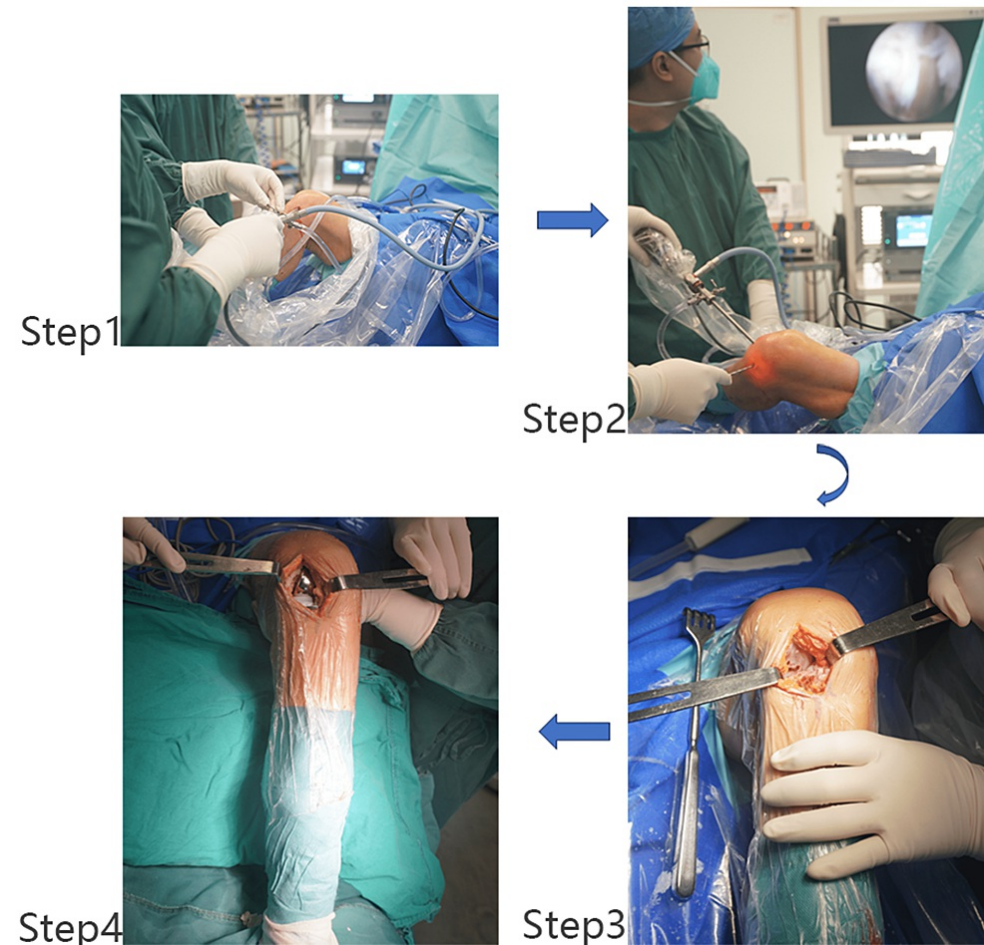
Arthroscopy and UKA procedures Step 1: Supine positioning and general arthroscopy setup were used. Step 2: the cartilage of each compartment and ACL function were the predominant factors influencing the surgical decision on whether to conduct UKA or TKA. The arthroscopy procedure also included the excision of synovium, removal of loose body, smoothening the bone surface, and reshaping and repairing the meniscus. Steps 3 and 4: The MIS approach to perform UKA. The arthroscopy medial working approach involved an incision. Extending the medial portal can be utilized as the surgical incision for minimally invasive UKA ACL: anterior cruciate ligament; MIS: minimally invasive surgery; TKA: total knee arthroplasty; UKA: unicompartmental knee arthroplasty

All surgeries were performed by senior surgeons after a thorough examination of each patient. The decisions on the surgical method of knee arthroplasty and whether to perform arthroscopic treatment were based on the clinical characteristics of each patient. Patients with single-compartment radiographic changes, multicompartment symptoms, ACL injuries, or a history of meniscus tears were advised to undergo the arthroscopy procedure. The surgery plan was discussed among all senior surgeons before surgery, and the final decision was made by the attending doctor. For patients who did not undergo arthroscopy procedures, the surgical decision was based on the surgeon’s experience. In those who underwent arthroscopy, all compartments of the articular joint were examined, and the tension of the ACL was assessed with a hook. The arthroscopy procedure included excision of synovium, removal of loose bodies, smoothening the bone surface, and reshaping and repairing the meniscus. The cartilage condition in each compartment was the predominant factor influencing the surgical decision on whether to conduct UKA or TKA (Figure 1). All implants were cemented. The TKA surgery utilized a midline incision with a medial parapatellar approach and a fixed-bearing polyethylene liner, while the UKA procedure used a minimally invasive surgery approach and a mobile-bearing polyethylene liner [10]. A drainage tube was placed for 24 hours. Active contraction of quadriceps femoris muscle and ankle exercises were started immediately after the surgery, while passive knee movement on continuous passive motion began 24 hours later. Patients were allowed to start partial weight-bearing with crutches three days after the operation, while full weight-bearing was permitted one week after the operation.

Clinical information and outcomes

Demographic and clinical data were collected during the inpatient stay and included patient age, gender, blood transfusion status, length of inpatient stay, and postoperative complications. Symptoms and radiographic imaging findings were collected during follow-up, either by phone or onsite physical examination. Radiographic data, including long-leg pictures as well as anterior-posterior and lateral pictures, were evaluated based on the hip-knee-ankle (HKA) angle. The functional abilities were assessed in the immediate postoperative period and further at 3, 6, 12, and 24 months by using the Western Ontario and McMaster Universities Osteoarthritis Index (WOMAC), Knee Society Score (KSS), Knee Society Functional Score (KSFS), and Forgotten Joint Score (FJS) [11].

Statistical analysis

Continuous variables were compared using the Student’s t-test while discrete variables were compared using the chi-square test. All tests were two-sided, and p-values <0.05 were considered statistically significant. All statistical analyses were performed on R version 4.2.1.

## Results

A total of 429 patients who underwent knee arthroplasty were included in our study. Among them, 38 patients underwent intraoperative arthroscopy, with 30 undergoing UKA and eight undergoing TKA. However, 391 patients did not undergo joint arthroscopy, including 17 who underwent UKA and 374 who underwent TKA.

In our study, we found that patients who underwent an arthroscopy were more likely to undergo UKA (78.9%) compared to those who did not undergo an arthroscopy procedure (4.3%, p<0.05). Among those who underwent UKA, no differences were observed in terms of age, gender, BMI, preoperative HKA angle, and operative time between the arthroscopy + UKA and UKA-only groups (Table 1). The average length of hospital stay among patients who underwent arthroscopy (12.10 ±3.70 days) was significantly lower than those without arthroscopy (17.35 ±3.52 days). Neither blood transfusions during hospitalization nor any postoperative complications were recorded in either group. At the two-year follow-up after surgery, we did not observe a significant difference in HKA (p=0.07) between the arthroscopy + UKA group (175.73 ±2.24) and the UKA-only group (174.12 ±3.04).

**Table 1:** Characteristics of patients undergoing UKA based on the application of arthroscopy *Statistically significant BMI: body mass index; HKA: hip-knee-ankle; SD: standard deviation; UKA: unicompartmental knee arthroplasty

Variable	UKA + arthroscopy	UKA-only	P-value
Gender, n (%)			0.08
Male	3 (10%)	6	
Female	27 (90%)	11	
Age, years, mean ±SD	67.73 ±7.78	65.47 ±7.20	0.32
BMI, kg/m^2^, mean ±SD	25.14 ±2.01	24.69 ±1.53	0.40
Operative time, minutes, mean ±SD	188.47 ±56.65	195.00 ±50.33	0.69
Length of hospital stay, days, mean ±SD	12.10 ±3.70	17.35 ±3.52	0.03*
Pre-surgery HKA angle, mean ±SD	176.03 ±1.13	176.18 ±2.13	0.80
Post-surgery HKA angle, mean ±SD	175.73 ±2.24	174.12 ±3.04	0.07

Regarding the WOMAC score, no differences were observed for postoperative WOMAC pain, WOMAC stiffness, and WOMAC functional scores between the two groups (p>0.05) (Table 2). Although we did not find any difference in KSFS between the arthroscopy + UKA group (89.17 ±3.47) and the UKA-only group (89.35 ±2.55, p=0.83), a significantly higher KSS score was observed in the arthroscopy + UKA group (88.77 ±5.09) compared to the UKA-only group (85.53 ±5.11, p=0.04).

**Table 2:** Comparison of pre and postoperative KSS, KSFS, and WOMAC scores in patients who underwent UKA based on the application of arthroscopy *Statistically significant KSFS: Knee Society Functional Score; KSS: Knee Society Score; SD: standard deviation; UKA: unicompartmental knee arthroplasty; WOMAC: Western Ontario and McMaster Universities Osteoarthritis Index

	Pre-surgery				Post-surgery		
	UKA + arthroscopy, mean ±SD	UKA-only, mean ±SD	P-value		UKA + arthroscopy, mean ±SD	UKA-only, mean ±SD	P-value
KSS	59.23 ±8.53	61.35 ±9.55	0.45		88.77 ±5.09	85.53 ±5.11	0.04*
KSFS	35.73 ±11.30	38.29 ±11.06	0.45		89.17 ±3.47	89.35 ±2.55	0.83
WOMAC							
Pain	14.37 ±2.36	15.82 ±3.00	0.10		9.93 ±2.82	9.77 ±2.68	0.84
Stiffness	6.27 ±1.08	5.59 ±1.176	0.06		2.93 ±0.74	3.06 ±0.90	0.63
Functional score	57.27 ±6.19	61.41 ±9.82	0.49		39.17 ±5.24	40.59 ±6.68	0.84
Total	77.9 ±7.27	82.82 ±10.43	0.10		52.03 ±6.04	53.41 ±6.83	0.49

Regarding FJS, the overall score was significantly higher in patients undergoing arthroscopy (44.6 ±4.20) than those without (42.05 ±3.58, p<0.05) (Table 3). A similar pattern was also observed in the subgroup scores. The FJS subgroup scores were significantly higher in patients undergoing arthroscopy than those without, including in terms of stair climbing (3.73 ±0.98 vs. 3.06 ±1.09, p=0.04), standing up from a low-sitting position (4.10 ±0.92 vs. 3.18 ±0.95, p=0.02), and playing their favorite sports (3.93 ±0.74 vs. 3.24 ±1.35, p=0.03).

**Table 3:** Comparison of postoperative FJS at the last follow-up in patients undergoing UKA based on the application of arthroscopy *Statistically significant FJS: Forgotten Joint Score; SD: standard deviation; UKA: unicompartmental knee arthroplasty

Item	UKA + arthroscopy, mean ±SD	UKA-only, mean ±SD	P-value
Awareness in bed at night	3.80 ±1.03	3.88 ±0.99	0.79
Awareness when sitting on a chair for more than 1 hour	3.50 ±0.94	3.71 ±0.92	0.47
Awareness when you are walking for more than 15 minutes	3.47 ±1.07	3.47 ±1.01	0.99
Awareness when bathing	3.67 ±1.06	3.47 ±1.18	0.56
Awareness when driving a car	3.63 ±1.10	3.59 ±1.06	0.89
Awareness stair climbing	3.73 ±0.98	3.06 ±1.09	0.04*
Awareness when walking on uneven ground	3.70 ±1.02	3.59 ±1.33	0.75
Awareness when standing up from a low-sitting position	4.10 ±0.92	3.18 ±0.95	0.002*
Awareness when standing for more than 1 hour	3.80 ±1.03	3.94 ±0.75	0.62
Awareness when doing household work	3.60 ±1.04	3.35 ±1.11	0.45
Awareness when trailing	3.67 ±1.15	3.59 ±1.00	0.82
Awareness during favorite sporting activities	3.93 ±0.74	3.24 ±1.35	0.03*
Total	44.6 ±4.20	42.06 ±3.58	0.03*

## Discussion

In our study, we first investigated the impact of arthroscopy on the surgical decision-making process regarding whether to perform UKA or TKA. We found that surgeons were more likely to choose UKA in patients who underwent arthroscopy. In line with the guidelines and consensus, patients with only classical symptoms of unicompartmental arthritis and a functional ACL were advised to undergo UKA. TKA was recommended when patients had multicompartmental symptoms or any sign of ACL function deficiency [5]. However, other factors, such as previous ACL injuries and meniscus tear history, might also be related to the severity of intra-articular lesions and therefore influence the surgical decision on whether to perform UKA or TKA. To comprehensively evaluate the lesion severity and joint pathology, arthroscopy was performed to guide the surgical decision-making. For instance, UKA would be performed in those with a functional ACL, regardless of whether they had multicompartmental symptoms or not.

Compared to TKA, UKA preserves the lateral compartment and ACL, resulting in better knee proprioception and improved functional outcomes for sports activities [12,13]. However, strict criteria related to indications limit its application. In the past, ACL-deficient knee used to be considered a contraindication for UKA [14]; however, recent studies have shown potential, particularly in cases with typical anteromedial knee patterns [15,16]. Comprehensive examination and treatment of the articular joint through arthroscopy provide valuable information on the severity of joint lesions, preventing ambiguity in surgical indication and making it possible to perform the UKA in patients with ACL deficiency [8,9,17,18]. In addition, arthroscopy also demonstrates superior diagnostic performance over conventional MRI. MRI has limited diagnostic performance in assessing ACL function and might overestimate the severity of ACL deficiency in OA patients [19,20]. Additionally, MRI may overestimate the cartilage damage in the lateral condyle and underestimate it in the medial condyle [21]. Arthroscopy overcomes these limitations, serving as the gold standard for diagnosing intra-articular knee pathology, by precisely evaluating ACL tension and blood supply. It enables comprehensive exploration of all articular cavities and thorough assessment of joint injuries, expanding the application of minimally invasive UKA. Moreover, knee pain has been found to be related to inflammation that is difficult to detect with radiography [22,23]. Arthroscopy-assisted procedures, such as excision of the synovial membrane, removal of the corpus liberum, brisement of the patellar retinaculum, meniscus repair, and chondroplasty, could reduce joint pain and cartilage wear in OA patient [24,25].

Although some studies have suggested that knee replacement shortly following arthroscopy might be associated with poorer outcomes and a higher risk of complications [26,27], the evidence remains inconclusive. In our study, we found no significant differences between the UKA-only and UKA + arthroscopy groups regarding operative time, blood transfusion, and postoperative complications. In addition, our results indicated that the minimally invasive arthroscopy did not explicitly increase the length of inpatient stay. Although a shorter hospital stay was observed in the UKA + arthroscopy group in our study, caution should be exercised in interpreting this result.

We also attempted to evaluate the impact of arthroscopy on patient outcomes. To provide a comprehensive assessment of postoperative functional recovery and personal satisfaction, we used multiple scales, including HKA, KSS, KSFS, and FJS scales [28]. At the two-year follow-up after surgery, we observed an improvement in alignment correction in patients who underwent UKA, regardless of whether arthroscopy was performed or not. A similar result was observed for KSFS. However, patients with additional arthroscopy procedures had higher FJS scores and better rehabilitation with regard to sports activities than patients with UKA only. Arthroscopic surgery might lead to less impairment in proprioception and postural control compared to conventional UKA. Liu et al. reported that arthroscopic evaluation is valuable in the determination of UKA indication and showed improved outcomes in OA patients [7]. Apart from its conventional indication of degenerated knee diseases [29], previous reports have also suggested favorable outcomes with arthroscopic surgery in patients who underwent arthroplasty [30]. Consistent with these findings, our study also suggests that arthroscopy as an assistive procedure might be associated with better outcomes in patients undergoing UKA.

Our study has a few limitations. Primarily, the study focused specifically on single compartmental OA patients with multicompartmental symptoms and a history of previous knee injuries. While these stringent inclusion criteria allowed us to eliminate the impact of other prognosis factors and explicitly explore the potential impact of arthroscopy on prognosis in OA patients, our findings may be limited in terms of generalizability. Secondly, the relatively small sample size and lack of external validation also warrant caution in generalizing our results. Third, although the senior surgeons conducted a comprehensive assessment and thoroughly reviewed the operation indication, we could not rule out the effect of personal preference influencing the selection of the operation procedure. Hence, multi-center studies with larger participant cohorts are warranted to validate our findings.

## Conclusions

Based on our findings, the superior inspection and diagnostic performance of arthroscopy enables a comprehensive assessment of whether the stringent indications for UKA are met in patients with OA. Simultaneous arthroscopy performed with UKA is associated with a favorable prognosis in OA patients, even among those with multicompartmental joint symptoms. Our results demonstrate that arthroscopy could provide reliable evidence on surgical decision-making and improve knee functional outcomes in OA patients.
